# Using technology to provide individualized respite for caregivers in rural communities

**DOI:** 10.3389/frhs.2025.1575763

**Published:** 2025-05-15

**Authors:** Linda Weiss, Ann Battaglia, Sheaba Daniel, Ashley Conti, Foram Jasani, John Tyler

**Affiliations:** ^1^ Center for Evaluation and Applied Research, The New York Academy of Medicine, New York, NY, United States; ^2^ Healthy Community Alliance, Gowanda, NY, United States; ^3^ The Philanthropic Initiative, Boston, MA, United States

**Keywords:** caregivers, respite, older adults, technology, rural health

## Abstract

**Introduction:**

Caregiving for older adults—by family and friends—is an essential component of the U.S. health and long-term care systems. Though often rewarding, caregiving is associated with higher rates of poor health. Respite is valued by caregivers and associated with positive outcomes; however, access is limited. Exhale—The Family Caregiver Initiative, known as Exhale, was established to support the development of respite programming consistent with local priorities.

**Methods:**

Exhale respite programs participate in an evaluation, which includes caregiver assessments. This paper focuses on results from one Exhale-supported program, Caregiver Tech Solutions (CTS). CTS provides digital technology and coaching to caregivers in rural New York, offering an alternative to place-based respite and allowing caregivers to achieve short breaks from tasks or worries within their homes, on their terms.

**Results:**

Evaluation findings show that most CTS caregivers were children of the care recipients and cared for someone age 75+. Most had not participated in a respite program previously. Significant increases in respite were reported at follow-up: 25% of participants reported respite “every day or almost every day” at follow-up, compared to 12% at baseline. There were also significant declines in caregiver burden.

**Conclusions:**

CTS is not unique in use of electronic resources; however, literature remains sparse. Furthermore, pairing technology with coaching is uncommon. Finally, the outcomes demonstrated by CTS counter perceptions that technology-based solutions are not appropriate for older adults or in rural communities. Rather, flexible programs such as CTS represent a promising approach to addressing the needs of rural caregivers.

## Introduction

Caregiving for older adults—by family, friends, and neighbors—is an essential component of the health and long-term care systems in the U.S.; caregiving makes it possible for millions of people to age at home rather than in congregate settings and reduces overall healthcare costs (1). According to the National Academies of Science, Engineering, and Medicine, at least 17.7 million individuals in the U.S. are caregivers of family members ages 65 and older (2).

Caregiver responsibilities may be time intensive (3), complex, and wide ranging. They commonly include medical tasks, navigation and coordination of care, household chores, financial management, and support with activities of daily living (1, 3, 4). Though often emotionally rewarding, caregiving is associated with higher rates of social isolation, depression, anxiety, and chronic disease; increased healthcare costs; and increased mortality (4, 5–10). Caregiving may be particularly challenging in rural areas. Formal services, including professional caregivers, home care, and other supportive services are less available; and travel to services that do exist is more costly and difficult (11, 12).

Respite, often referred to as a “break” from caregiving (13) is highly valued by caregivers (5) and associated with positive outcomes (14) including reduced stress (5), increased confidence in ability to continue to provide care (15), and improved quality of life (16). However, a relatively small proportion of caregivers who may benefit currently use respite services due to lack of availability or awareness, poor fit (e.g., high cost), and rigid models of respite that may not meet expectations or needs of all populations, including programs offered only at a fixed location and a set time (10, 13, 17, 18).

Recognizing the critical role caregivers play in the lives of older adults and an unmet need for respite, *Exhale—The Family Caregiver Initiative*, also known as Exhale, was established in 2019 to provide financial and technical support to organizations in Western New York State and Southeast Michigan seeking to develop or expand respite programming consistent with community priorities; the initiative is managed by The Philanthropic Initiative (TPI) (19). To date, 27 respite programs have received multi-year funding through Exhale, involving well over 100 partner organizations. Models and services vary and include place-based respite (e.g., drop-off programs), technology-based respite, wellness classes, caregiver education, social and recreational opportunities, and practical assistance within the home (e.g., home remediation to meet the needs of older adults with limited mobility, household chores). Through TPI, Exhale organizations have access to a variety of supports, including a learning collaborative, training and assistance with project management and creative problem solving, and assistance with communications (e.g., branding, outreach).

As described in the section below, Exhale includes a mixed-method, multilevel, pragmatic evaluation, designed to support the implementation of Exhale and to inform the expansion of innovative respite models more generally. The evaluation has four main objectives, to: (1) assess changes in access to, and use of, respite among caregivers of older adults; (2) build the evidence base—including indicators such as participant satisfaction and outcomes—to better support respite needs and innovative respite models; (3) document the development, implementation and significance of new collaborative partnerships; and (4) examine the Exhale model and component parts. This paper focuses on the first two objectives: assessing access and use of respite, as well as perceptions and outcomes. Its intended audience is key stakeholders (e.g., practitioners, policy makers, payors, researchers, and others involved in aging and/or respite services more broadly) to support replication, sustainability, and further investigation of innovative models, as appropriate.

## Materials and methods

All Exhale programs are required to participate in the above-referenced multisite evaluation. This paper focuses on one set of findings from the evaluation: quantitative caregiver-specific results from one of the initial Exhale-supported programs, Caregiver Tech Solutions (20).

### Intervention

Developed and first funded in 2020 as part of the first Exhale cohort, Caregiver Tech Solutions is a program of the Healthy Community Alliance, a rural health network. Caregiver Tech Solutions focuses on the use of digital technology to help caregivers of older adults (defined as age 55 and older) who are living in any of three rural New York State counties achieve short breaks from tasks and worries. Offering an alternative to traditional place-based programs, Caregiver Tech Solutions offers caregivers who meet the above criteria respite periods at home and on terms that suit them. Program resources also focus on reducing caregiver strain, learning self-care techniques, and solving caregiving issues that may arise. The Caregiver Tech Solutions model, developed with input from caregivers, consists of:

• completion of an individualized assessment, “What Matters Most,” to identify caregiving-related needs, values, and priorities, which may include—for example—time management, recreation, socialization, stress reduction, and care recipient safety;
• eight one-on-one coaching sessions with program staff to identify, design, and support the use of technology products intended to provide caregiver respite;
• technology-based products to meet individual needs, including indoor and outdoor security cameras; tablets, smart watches, speakers, and plugs; window and door alarms; voice assistants; digital medical reminders; Bluetooth trackers; robotic vacuums; animatronic animals; and video doorbells; and
• follow-up to assess program outcomes and to facilitate external referrals to meet additional caregiver needs.

Outreach and recruitment of participants is conducted using flyers distributed at community events, advertisements in local periodicals, presentations to referral partners, and word of mouth. The program offers resources that do not require an internet connection, as well as resources that do, meaning that participation is open to those with and without broadband and WIFI access. Caregiver Tech Solutions is staffed by two part-time coaches, both with prior experience providing case management services to older adults; one coach is based at the Healthy Community Alliance and one at a county Office for Aging.

### Evaluation design and methods

Exhale is being evaluated by the Center for Evaluation and Applied Research at The New York Academy of Medicine (NYAM). The evaluation includes caregiver assessment surveys administered by each of the funded projects, as well as qualitative interviews with project leadership and their partners, Exhale leadership, and caregivers. Assessment instruments were developed by NYAM evaluators in collaboration with TPI. The evaluation protocol and related materials were reviewed and approved by NYAM's Institutional Review Board.

### Data collection

This paper reports on findings from caregiver assessment surveys conducted at baseline (i.e., enrollment in the program) and 3-months from baseline, an interval of time that allowed for completion of the eight coaching sessions, as well as use of the resources made available through the program. Baseline and 3-month follow-up assessments were linked by a unique study ID. The baseline assessment includes close-ended questions on basic demographics (e.g., age, gender, race or ethnicity), health of the caregiver and care recipient, caregiving responsibilities, and prior and current respite experience. The three-month follow-up assessment includes the same questions as the baseline, with the exception of questions on demographics and prior respite experience, which were excluded. The follow-up assessment also includes questions on continued engagement with the program and continued use of program resources, satisfaction with the program, and perceived impact. In addition to the caregiver assessments, participants completed the ARCHANGELS (21) Caregiver Intensity Index (CII) at the same two points in time. The CII is a caregiver survey that assesses the intensity of a caregiving situation and has been used in practice (e.g., to identify caregiver need for services) and in research (22–25). Once complete, the CII provides a score (range 0–100), grouped as low (0–24), moderate (25–54), and high (55–100). The CII also lists specific caregiving intensity buffers (e.g., workplace support) and drivers (e.g., financial burdens), to help caregivers reduce intensity. CII data were linked to caregiver assessment data with the unique study ID.

Caregiver assessment and CII data were completed by participants during one-on-one sessions with the program coaches, in person, by phone, or on a video call—allowing for participation of individuals with and without computer skills and internet access. Data were collected from October 2021 (the start of the Caregiver Tech Solutions program) through September 2024.

### Data management and analysis

Data were electronically submitted to NYAM for cleaning, management and analysis using STATA SE (version 15; College Station, TX). Descriptive statistics (means and proportions) were generated for all variables. The Wilcoxon matched-pairs signed rank test and paired *t*-test were used to test for statistically significant change (*p* < .05) from baseline to the 3-month follow-up.

## Results

During the study period, 129 caregivers enrolled in the program; all completed baseline assessments. One hundred and twenty-one participants (94%) completed the program and a 3-month follow-up assessment. Reasons for program and assessment non-completion included lost to follow-up (*n* = 4 participants) and death of the care recipient or caregiver (*n* = 4 participants). Of the 121 participants with baseline and 3-month follow-up assessments, 102 completed CII assessments at both points in time.

Table 1 displays caregiver demographic and health information at baseline. Approximately 72% of caregivers were age 55 and older; approximately one-third (34%) were age 65 and older. Over 80% of caregivers were women, and most were white (96%). A little less than half were employed full- or part-time (42%); 40% were retired. Over 95% of the caregivers were insured: 51% had private insurance, 36% were insured by Medicare, and 12% were insured by Medicaid. Eighty-eight percent of the caregivers reported being in excellent, very good or good health.

**Table 1:** Caregiver demographics and health Status (*N* = 129).

Caregiver characteristics	*n* (%)	
Age (in years)		
18–54	35	(27%)
55–64	49	(38%)
65–74	38	(29%)
75+	7	(5%)
Gender		
Woman	105	(81%)
Man	24	(19%)
Race or ethnicity^a^		
White	124	(96%)
Hispanic or Latinx	2	(2%)
Black or African American	1	(1%)
Indigenous American or Alaska Native	1	(1%)
Prefer not to answer/Missing	1	(1%)
Current health insurance^a^		
Medicare	46	(36%)
Medicaid	15	(12%)
Other health insurance (includes private/commercial)	66	(51%)
Not insured	4	(3%)
Health status		
Excellent	14	(11%)
Very good	39	(30%)
Good	60	(47%)
Fair	15	(12%)
Poor	0	(0%)
Missing	1	(1%)

^a^ Multiple responses permitted.

Table 2 displays participants’ experience with caregiving and respite programs. Most caregivers (60%) were the children of the care recipients; 27% were spouses. Approximately two-thirds (69%) cared for someone aged 75 or older. Most (92%) provided care in person; and over half (54%) lived in the same households as the care recipients. Most caregivers report providing help in a broad range of areas including health management (95%), social support (88%), transportation (87%), financial management (87%), shopping (86%), and cooking (84%) (data not shown).

**Table 2:** Caregiving and respite (*N* = 121).

Experience with caregiving and respite	Baseline	3-month follow-up
	*n* (%)	*n* (%)
Caregiver relationship to care recipient		
Child of care recipient	72 (60%)	N/A
Spouse or partner	33 (27%)	
Other relative	13 (11%)	
Friend	1 (1%)	
Neighbor	1 (1%)	
Missing	1 (1%)	
Age of care recipient		
55–64	10 (8%)	N/A
65–74	28 (23%)	
75–84	39 (32%)	
85+	44 (36%)	
Care location		
In person (live in the same household)	65 (54%)	72 (60%)
In person (live in separate households)	45 (37%)	40 (33%)
Both—in person and remote	9 (7%)	9 (7%)
Remote only	1 (1%)	0 (0%)
In Person (not specified if same or separate households)	1 (1%)	0 (0%)
Caregiver experience with respite programs		
Never before participated in a respite program	101 (83%)	N/A
Participated in another respite program in the past, not currently	7 (6%)	
Currently participating in a respite program in addition to this one	12 (10%)	
Missing	1 (1%)	
In the past month, frequency of having a period of rest or relief from caregiving responsibilities^a^		
Everyday or almost everyday	14 (12%)	30 (25%)
At least once a week but not everyday	47 (39%)	58 (48%)
At least once a month but not every week	17 (14%)	29 (24%)
I had no opportunities for rest or relief	38 (31%)	4 (3%)
Don't know	4 (3%)	0 (0%)
Prefer not to answer/Missing	1 (1%)	0 (0%)

^a^ *p*-value < 0.05 in Wilcoxon matched-pairs signed-rank test.

The majority of caregivers (83%) had not participated in a respite program prior to their engagement with Caregiver Tech Solutions. Significant increases in rest or relief (i.e., respite) from caregiving were reported at the 3-month follow-up. Twelve percent of participants reported rest or relief “every day or almost every day” at baseline, compared to 25% at the follow-up. At baseline, 31% reported no opportunities for rest or relief, compared to 3% at the 3-month follow-up. These differences were statistically significant with *p* < 0.05 on the Wilcoxon signed rank test for matched pairs.

**Figure 1:**
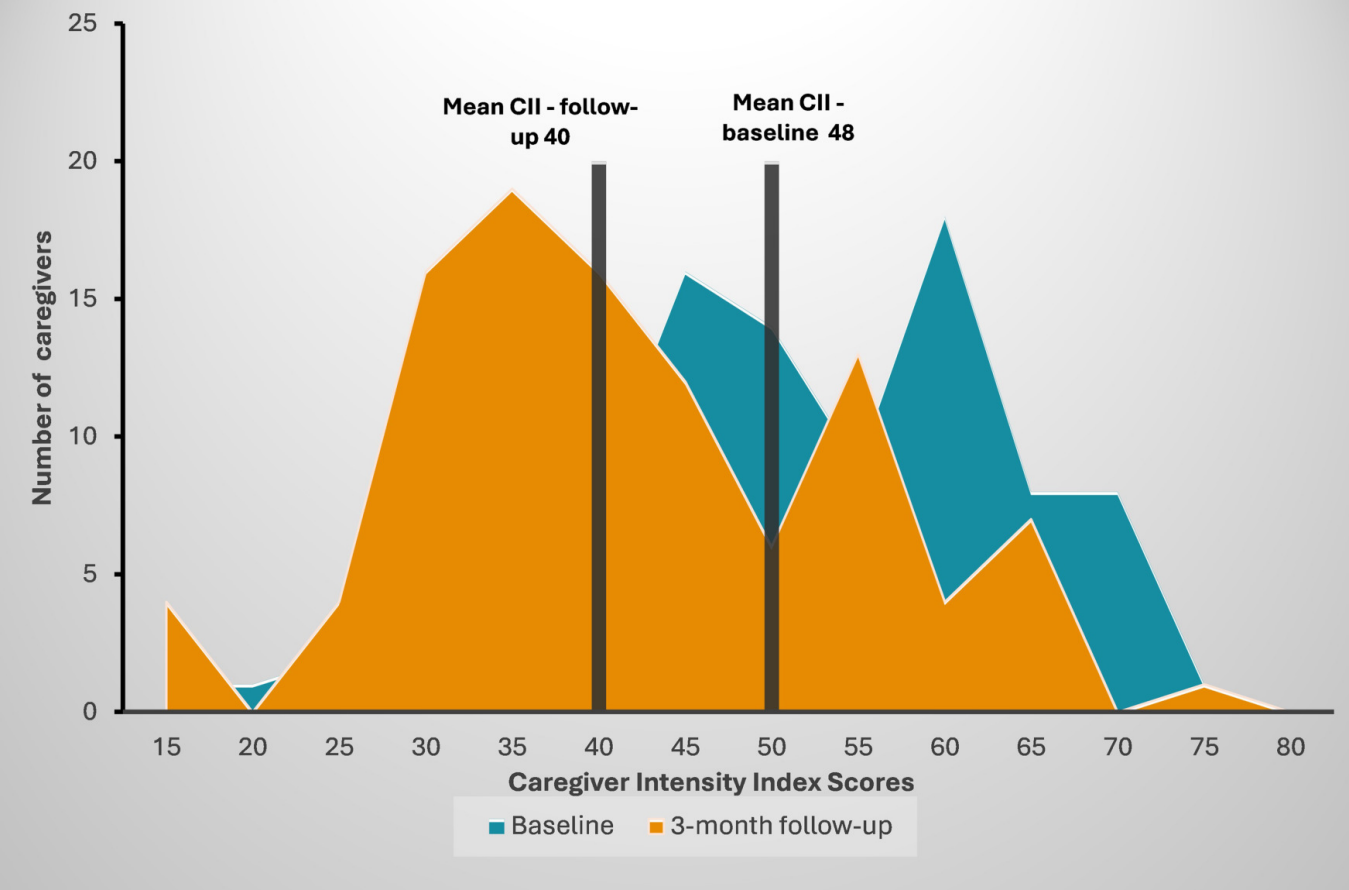
Caregiver intensity index scores at baseline and 3-month follow-up (*N* = 102).

As displayed in Figure 1, there were statistically significant declines in caregiver burden, as measured by the Caregiver Intensity Index (CII) scores at baseline and the 3-month follow-up. Over one-third of caregivers displayed high caregiver intensity at baseline as compared to 14% at 3-month follow-up. The average caregiver intensity score decreased from 48 to 40 on a 0–100 point scale (*p* < 0.01 in paired *t*-test).

As shown in Table 3, most participants had positive perceptions of Caregiver Tech Solutions at the 3-month follow-up. They reported that “because of the program” they learned about new opportunities to take a break from caregiving (99%), have new resources to support them as a caregiver (98%), feel supported knowing they have ready access to supports to take a break when needed (98%), and have new opportunities for taking a break from caregiving (95%). Sixty percent of participants indicated that they had connected to other caregivers through Caregiver Tech Solutions.

**Table 3:** Perceptions of program participation at 3-month follow-up (*N* = 121).

Program impacts	*n* (%)
Because of this program …^a^	
I know about new opportunities for taking a break from caregiving	120 (99%)
I have new resources that support me as a caregiver	119 (98%)
I feel supported knowing that I have ready access to supports that allow me to take a break from caregiving when needed	119 (98%)
I have new opportunities for taking a break from caregiving	115 (95%)
The person I care for has new recreational or social opportunities	79 (65%)
I have connected to other caregivers	72 (60%)

^a^ Multiple responses permitted.

## Discussion

The evaluation of Caregiver Tech Solutions shows several positive results. Survey data indicate that the program has successfully engaged caregivers that are new to respite: 83% of participants had never before participated in a respite program. The relatively high number of participants working full or part-time (42%) suggests that a model like the one used by this program may be particularly appropriate for employed caregivers, who often face significant strain (26) but may be unable to access or benefit from respite opportunities offered during regular working hours. Furthermore, participants reported both increased respite over time and multiple associated benefits. The proportion of participants reporting respite at least weekly increased from 51% at baseline to 73% at the 3-month follow-up; daily respite increased from 12% at baseline to 25% at 3-months. Virtually all participants reported having new resources (98%) and new opportunities (95%) for taking a break from caregiving. There were statistically significant decreases in caregiver burden from baseline to three months, although burden was considered “moderate” at both points in time. That caregiver burden was not reduced more substantively may reflect declines in health among care recipients, which can lead to greater caregiving responsibilities, even within relatively short periods of time. It may also reflect the fact that periods of respite do not fully remove the many challenges of caregiving and that caregivers and care recipients may still have unmet needs for other concrete supports and services.

Caregiver Tech Solutions is not unique in facilitating respite through digital and other electronic resources (27); however the literature—particularly as related to outcomes—remains sparse (28) and focused more on models that use technology to access respite rather than provide it (29). Furthermore, pairing technology with comprehensive and individualized coaching is relatively uncommon, despite its value in ensuring optimal selection and use of digital resources—and the opportunities coaching offers to more fully address caregiver needs and priorities. For example, caregivers most concerned with the safety of those they care for might be offered in-home cameras, while those feeling isolated due to caregiving responsibilities might opt for a tablet to remotely connect with friends and family members. Finally, the level of engagement and positive outcomes demonstrated by Caregiver Tech Solutions counters perceptions that technology solutions would not be appropriate for older adults or in rural communities. Although disparities in broadband and computer access remain (30–32) there is evident capacity and interest. Rural communities have proportionately more older adults than urban and suburban communities and fewer services to support them (11). Flexible programs such as Caregiver Tech Solutions represent a promising approach to addressing the needs of rural caregivers.

This study has several limitations. The individualized nature of services means that the specific supports and devices participants receive are likely to differ. This may dilute effects (e.g., if some individuals receive fewer supports) and make replication somewhat challenging. In addition, the analysis presented here represents one program within the multisite Exhale evaluation, which includes a range of models and organizational capacities. Given the need to include all programs in the evaluation, and concerns about data quality and burden, we were unable to gather the range of data that might have been included in a single site research study.

Despite limitations, we feel the findings make an important, pragmatic contribution to the literature on effective respite programming. Findings demonstrate that the program successfully leverages the benefits of technology to provide individualized in-home and on-demand respite consistent with the values, priorities, and schedule of participating caregivers. Without the need to travel to place-based services, respite can be frequent, brief, ongoing, and/or interspersed with daily activities. Future research should have greater focus on the Caregiver Tech Solutions model, and models that are similar in design, assessing engagement and outcomes associated with specific participant characteristics; types of technology and other services used; as well as service gaps, unmet needs, and areas for improvement. A more focused study with more comprehensive data collection may better guide replication and expansion and support the continued development of an evidence base for technology-focused respite services. In addition to continued research, the positive findings point to the potential for expansion in the future, particularly in rural areas that face barriers to place-based services, as well as a broadening of the scope of payor-supported respite, to ensure the viability of effective services.

## Data Availability

The raw data supporting the conclusions of this article will be made available by the authors, without undue reservation.
